# Lateralized modulation of cortical beta power during human gait is related to arm swing

**DOI:** 10.1016/j.isci.2024.110301

**Published:** 2024-06-17

**Authors:** Marzieh Borhanazad, Bernadette C.M. van Wijk, Annemieke I. Buizer, Jennifer N. Kerkman, Annike Bekius, Nadia Dominici, Andreas Daffertshofer

**Affiliations:** 1 Department of Human Movement Sciences, Faculty of Behavioural and Movement Sciences, Vrije Universiteit Amsterdam, Amsterdam 1081 BT, the Netherlands; 2 Amsterdam Movement Sciences, Rehabilitation & Development, Amsterdam, the Netherlands; 3 Institute for Brain and Behavior Amsterdam, Vrije Universiteit Amsterdam, Amsterdam, the Netherlands; 4 Department of Neurology, Amsterdam UMC Location University of Amsterdam, Amsterdam 1105 AZ, the Netherlands; 5 Department of Rehabilitation Medicine, Amsterdam UMC Location Vrije Universiteit Amsterdam, Amsterdam 1081 HZ, the Netherlands; 6 Department of Neurology and Neurosurgery, UMC Utrecht Brain Centre, Utrecht University, Utrecht 3584 CG, the Netherlands

**Keywords:** Neuroscience, Sensory neuroscience, Cognitive neuroscience

## Abstract

Human gait is a complex behavior requiring dynamic control of upper and lower extremities that is accompanied by cortical activity in multiple brain areas. We investigated the contribution of beta (15–30 Hz) and gamma (30–50 Hz) band electroencephalography (EEG) activity during specific phases of the gait cycle, comparing treadmill walking with and without arm swing. Modulations of spectral power in the beta band during early double support and swing phases source-localized to the sensorimotor cortex ipsilateral, but not contralateral, to the leading leg. The lateralization disappeared in the condition with constrained arms, together with an increase of activity in bilateral supplementary motor areas. By contrast, gamma band modulations that localized to the presumed leg area of sensorimotor cortex around the heel-strike events were unaffected by arm movement. Our findings demonstrate that arm swing is accompanied by considerable cortical activation that should not be neglected in gait-related neuroimaging studies.

## Highlights

• Beta and gamma band EEG activity are modulated at specific phases of the gait cycle
• Beta modulations localize to the sensorimotor cortex ipsilateral to the leading leg
• The lateralization of beta modulations relates to arm swing movement
• Gamma activity increases around heel strike in (pre-)SMA and the medial part of M1

## Graphical abstract

**Figure:**
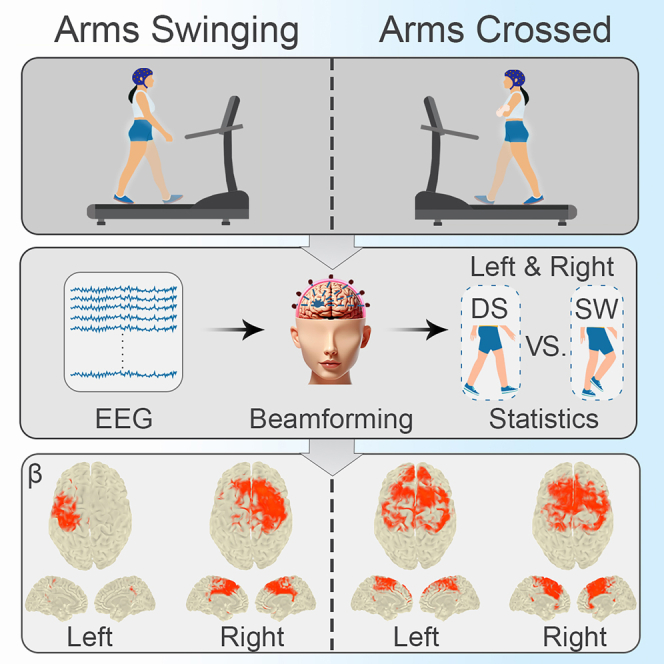

## Introduction

Human gait is a complex behavior requiring dynamic control of upper and lower limb movement. During walking, the arms move in opposition to the legs, in a back-and-forth motion. This arm swing behavior has been argued to be important not only because of biomechanical factors,^1^ but also because of its influence on leg activity due to neural coupling between arms and legs.^2^^,^^3^ Neuroanatomically, the spinal cord and distributed (sub)cortical regions are involved in the control of gait.^4^^,^^5^ Central pattern generators (CPGs) in the spinal cord play an important role in the generation of basic rhythmic locomotor patterns.^6^^,^^7^ In turn, the CPGs are influenced by input from supraspinal areas, where the cortex may be involved in the coordination and timing aspects of limb activation during the gait cycle.^8^ Gait phase-dependent modulations of cortical neural activity have been shown in several electroencephalography (EEG) studies. These modulations have been reported as changes in spectral power in theta (4–7 Hz), alpha (8–12 Hz), beta (13–30 Hz), and gamma (>30 Hz) frequency bands in distributed brain areas, particularly in the primary motor cortex (M1) and supplementary motor area (SMA) (see Delval et al.^5^ for review). However, how neural activity at these frequencies in different brain areas contributes to the activation of upper and lower limb muscles at specific times in the gait cycle is not fully understood.

The modulation of cortical power occurs around two gait events: heel-strike (HS) and toe-off (TO). These moments separate double support (DS) and swing (SW) phases (= single support) and indicate the onset of loading responses and push-off work. Arguably, the first is followed by a substantial balance demand and the latter subserves the forward locomotion. Event-related de-synchronization (ERD) of alpha/beta activity in the sensorimotor cortex has been observed prior to TO and during the SW phase, followed by beta/gamma event-related synchronization (ERS) at the end of the SW phase just before and during HS, and theta ERS during the DS phase and TO.^9^^,^^10^^,^^11^^,^^12^ Zhao and co-workers^13^ source-localized spectral power in different frequency bands across four phases of the gait cycle, and found alpha ERD and beta ERD/ERS to be lateralized in M1 during the SW and DS phases, and gamma power modulations to be present in medial motor areas. Different aspects of human walking have been associated with alterations in the magnitude of ERD/ERS patterns, often in multiple frequency bands. In general, greater theta ERS has been found during incline walking, loss of balance, and after sensorimotor perturbations.^11^^,^^14^^,^^15^ The modulations in alpha and/or beta power have been linked to gait stabilization,^14^^,^^16^^,^^17^ gait adaptation,^18^ and speed control.^19^^,^^20^^,^^21^ Gamma activity on the other hand, has been suggested to reflect sensorimotor processing during the timing of motion sequences during gait,^10^ and has been linked to gait-related attentional demand.^22^^,^^23^

The role of arm swing in gait has been extensively studied both in terms of biomechanics and muscular activity. It has been shown that this movement of the arm is not a passive motion^1^ and in fact influences gait stability.^17^^,^^24^ Consequently, any disruptions or abnormalities in arm swing, such as those caused by neurological conditions, can adversely impact gait stability, potentially leading to an increased risk of falling.^1^ Hence, a comprehensive understanding of the cortical contributions to arm swing movement is essential for gaining insights into the intricate interplay between the upper and lower body movements, further enriching our understanding of human locomotion and its underlying neural control. The modulation of EEG spectral power has also been observed in relation to the control of lower and upper limb movement during gait. By comparing gait with and without arm swing, Weersink and co-workers^12^^,^^25^ showed a significant reduction in alpha and beta power modulations in EEG electrodes over SMA when the arms were constrained. Instead, an increase in gamma power was found in electrodes covering the leg M1 area, which could be due to an increased need to maintain the cyclic movement pattern of the legs in the absence of arm swing. Modulations in the theta band proved to be less consistent within the gait phase and across electrodes. These studies however relied on sensor level analysis, which comes with inherent limitations in accurately localizing the precise underlying neural sources. To obtain a more detailed assessment of the cortical involvement in gait control, in this study, we sought to source localize brain activity in specific phases of the gait cycle, and to determine the effect of arm swing on this activity by letting participants walk on a treadmill with and without arm swing. We restricted our analyses to the beta and gamma frequency bands for which our hypotheses were most strongly supported by the literature. Specifically, we expected the lateralization of beta power modulations in M1 to be reduced when the arms are constrained, whereas gamma power in medial source locations might be increased as a possible compensation mechanism for the lack of arm swing.

## Results

**Figure 1:**
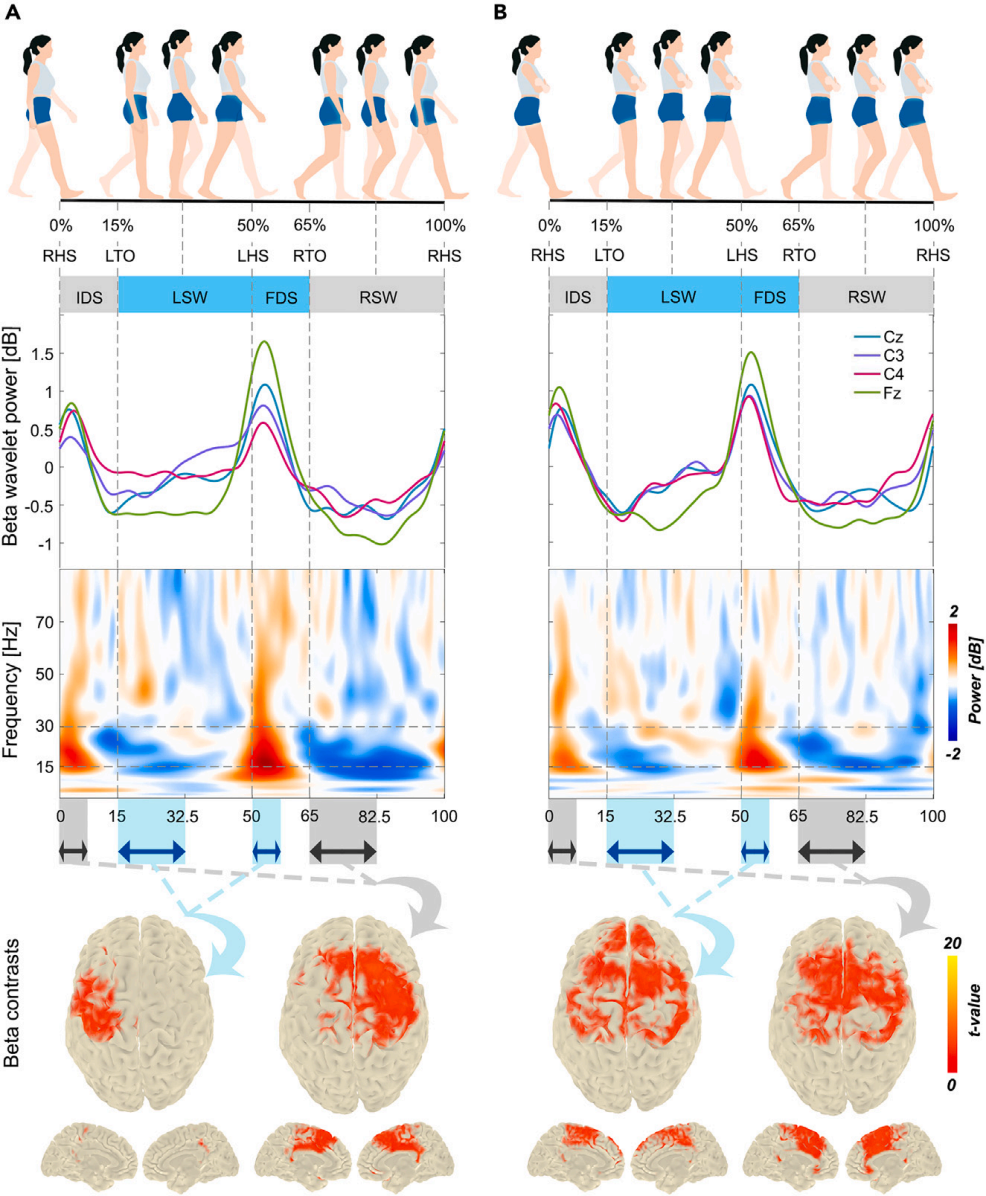
Time-frequency analysis and beamforming contrasts between early DS and SW phases in the beta band (A) *Arms swinging* condition. (B) *Arms crossed* condition. The average beta wavelet power is illustrated on top, and the time-frequency representations for electrode Cz are shown in the middle of each figure. The statistical contrast between beta beamformers (based on all 64 electrodes) in early DS and SW phases for each leg is illustrated at the bottom of the figure. The horizontal arrows indicate early (first half) DS and SW phases. *T*-values for voxels belonging to significant clusters (*p* < 0.0125; adjusted alpha level for four comparisons) are displayed after anatomical masking. Note the left-right symmetrical activation in the *arms crossed* compared to the *arms swinging* condition. DS, double support; SW, swing; IDS, initial double support; LSW, left swing; FDS, final double support; RSW, right swing.

To investigate the cortical involvement in arm swing during different phases of the gait cycle, we conducted an EEG study with a group of young adults. Participants were asked to walk on a treadmill with and without arm swing. We divided the gait cycle into four distinct phases, which we will hereafter refer to as right/left swing (R/LSW) and initial/final DS (I/FDS); please see Figure 1. We first analyzed the EEG signals at the sensor level using time-frequency analysis. Subsequently, we conducted source-level analysis using dynamical imaging of coherent sources (DICS) beamforming in both beta (15–30 Hz) and gamma (30–50 Hz) frequency bands. For each frequency band, we performed statistical comparisons between the DS and SW phases for each side of the body separately. For a more comprehensive understanding of our methodology, please refer to the STAR Methods section.

### Gait characteristics

Compared to walking with arm swing, walking with arms crossed resulted on average in larger stride durations but not in altered coefficients of variation; see Table 1. Specifically, the average duration of the LSW phase and of the two DS phases was larger in the *arms crossed* condition, but not for the RSW phase.

**Table 1:** Gait characteristics for walking with and without arm swing

Duration (s)	Walking with arms swinging		Walking with arms crossed		*p*-value	*p*-value
	Mean ± SD (s)	CV (%)	Mean ± SD (s)	CV (%)	Mean	CV
Stride	1.18 ± 0.07	5.33 ± 0.77	1.20 ± 0.06	6.08 ± 1.54	0.03	0.21
RHS to LTO (IDS)	0.18 ± 0.01	11.00 ± 1.52	0.18 ± 0.01	10.60 ± 1.63	0.02	0.59
LTO to LHS (LSW)	0.41 ± 0.03	5.49 ± 0.89	0.42 ± 0.03	6.93 ± 2.12	0.04	0.08
LHS to RTO (FDS)	0.18 ± 0.01	10.33 ± 1.91	0.18 ± 0.01	10.18 ± 1.53	0.02	0.86
RTO to RHS (RSW)	0.41 ± 0.03	5.61 ± 1.21	0.42 ± 0.03	6.79 ± 1.51	0.31	0.06

Mean durations and corresponding coefficients of variation (CV) are listed for the full stride and for each gait phase separately together with the resulting *p*-values from the paired *t-*tests between walking with arms swinging and arms crossed. IDS, initial double support; LSW, left swing; FDS, final double support; RSW, right swing.

### Time-frequency analysis at sensor level

The sensor level analysis was performed as a control analysis to compare with previous studies.^12^^,^^25^ Therefore, we only briefly describe our observations at the sensor level and report all subsequent statistical assessments at the source level.

**Figure 2:**
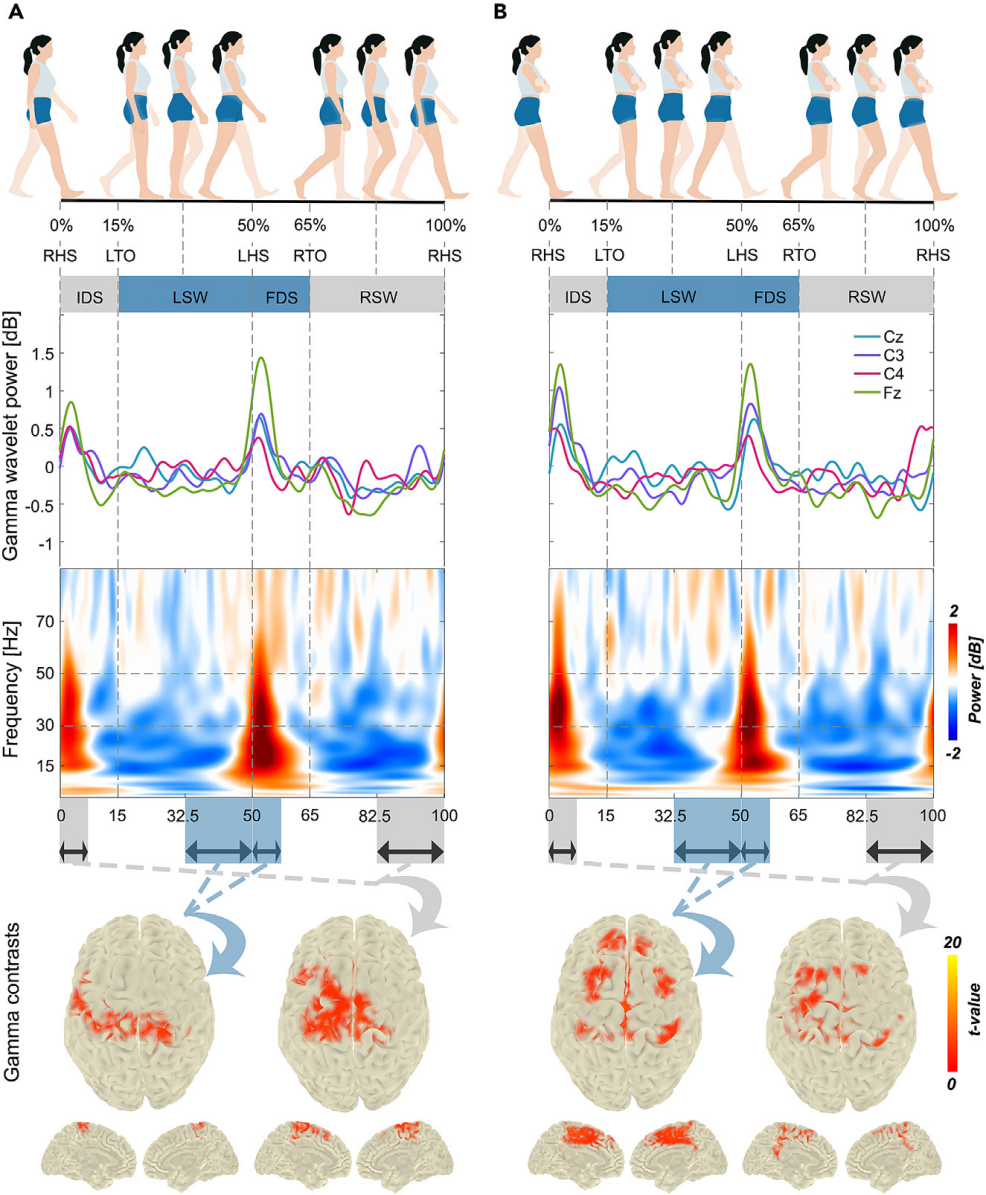
Time-frequency analysis and beamforming contrasts around the HS event in the gamma band (A) *Arms swinging* condition. (B) *Arms crossed* condition. The average gamma wavelet power is illustrated on top, and the time-frequency representations for electrode Fz are shown in the middle of each figure. The statistical contrasts between gamma beamformers (based on all 64 electrodes) in early DS and late SW phase (around HS event) for each leg is illustrated at the bottom of the figure. The horizontal arrows indicate early (first half) DS and late (second half) SW phase. *T*-values for voxels belonging to significant clusters (*p* < 0.0125; adjusted alpha level for four comparisons) are displayed after anatomical masking. HS, heel-strike; DS, double support; SW, swing; IDS, initial double support; LSW, left swing; FDS, final double support; RSW, right swing.

The average relative log wavelet power from electrodes Cz, Fz, C3, and C4 in both walking conditions are depicted in Figures 1 and 2 for the beta and gamma band, respectively. In addition, time-frequency spectra are shown in Figure 1 for electrode Cz, Figure 2 for electrode Fz, and Figures S1 and S2 for electrodes C3 and C4, respectively. In summary, the results revealed a clear pattern of ERD in the SW and ERS in the DS phase of each leg in both walking with and without arm swing, in particular in the beta band (Figure 1). However, the left-right lateralization of ERD/ERS beta modulations in the *arms crossed* condition appeared reduced compared to *arms swinging*, and was accompanied by an increase in the upper beta/lower gamma band in midline electrodes (Figure 2). In the following, we give a more detailed description of these observations.

#### Results from electrodes C3 and C4 (putative right and left arm)

In the *arms swinging* condition, the beta ERS in the DS phases was greater for the electrode ipsilateral to the leading leg compared to contralateral. In other words, the beta ERS for electrode C3 was greater during FDS (LHS-RTO) than during IDS (RHS-LTO), while for electrode C4, the beta ERS was greater during IDS than during FDS (see Figure 1 for average beta wavelet power; Figures S1 and S2 for the time-frequency spectrum). Power differences were also visible in the SW phases, with greater beta ERD in RSW compared to LSW for both electrodes. In the *arms crossed* condition, however, the beta ERS during the DS phases contralateral to the leading leg was greater compared to the *arms swinging* condition. As a consequence, electrodes C3 and C4 seemed to have equal beta power in both the DS and SW phases in the *arms crossed* condition; hence not showing a clear left-right lateralization.

#### Results from electrodes Cz and Fz (putative leg area and SMA)

In the *arms swinging* condition, both beta and gamma ERS in electrode Fz showed a difference in power between IDS and FDS, with slightly higher power in FDS, while these differences were reduced in the *arms crossed* condition, especially in the gamma band (Figure 2). This reduced difference was primarily due to an increase in ERS during the IDS phase, while the ERS in the FDS phase remained similar. Gamma band modulations were smaller for electrode Cz, suggesting a more frontal topography.

### Source localization

#### Beta frequency band

We first performed cluster-based permutation pairwise *t-*tests to identify the differences between early DS and SW phases. For the changes in source activity around the HS events, see Figure S1 in the supplementary material. By comparing early DS and SW phases in the *arms swinging* condition (Figure 1A, bottom row), we found significant activity in M1 and in premotor cortex in the hemisphere ipsilateral to the moving leg (*p* < 0.001). For the contrast on the right leg, additional significant activity (*p* < 0.001) was observed in bilateral SMA, mid and posterior cingulate cortex. In the *arms crossed* condition (Figure 1B, bottom row), however, the lateralized pattern was replaced by symmetric activity in the frontal cortex (*p* < 0.001). Again, only for the right leg contrast, activity was also observed in the cingulate cortex but more anteriorly compared to the *arms swinging* condition.

**Figure 3:**
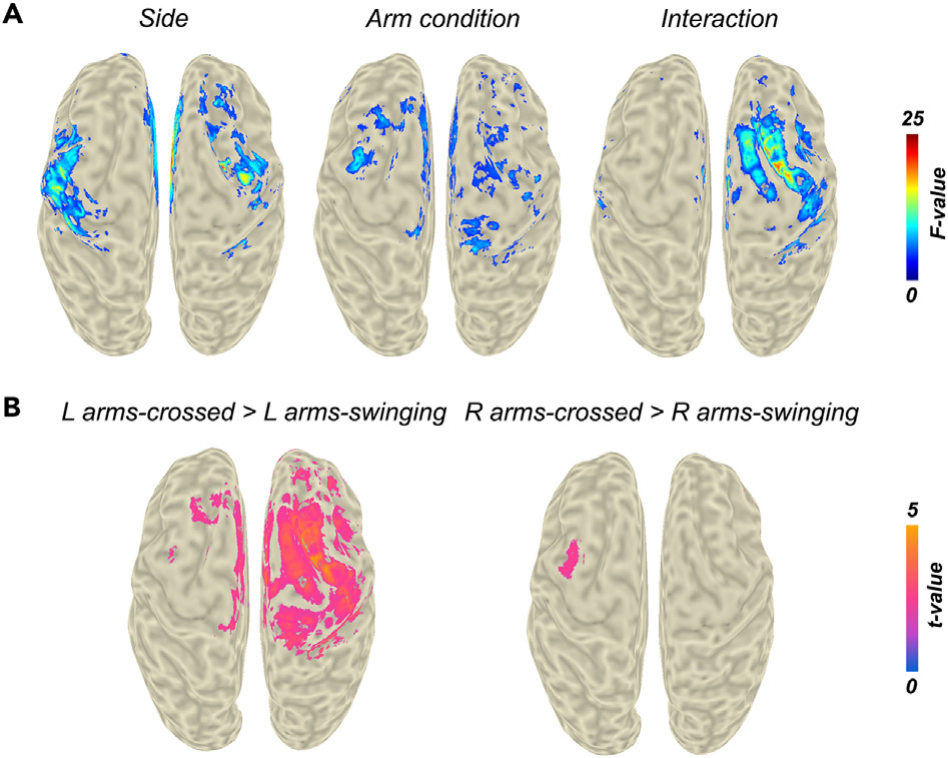
Statistical analysis of the beta band beamforming contrasts between early DS and SW phases Results indicate significant differences in the magnitude of the contrast between early DS and SW phases. (A) Two-way ANOVA with side (left vs. right leg) and arm condition (*arms swinging* vs. *arms crossed* condition) as the main factors. The maps were thresholded at *p* < 0.05. (B) Post-hoc *t*-tests for the main effect of arm condition. The maps were thresholded at *p* < 0.025, adjusted alpha level for two comparisons. DS, double support, SW, swing; R, right; L, left.

The subsequent ANOVA resulted in a significant main effect of body side (Figure 3A, left panel) with extensive clusters in anterior cingulate cortex, left (pre-)motor cortex, right (pre-)motor cortex, and a significant main effect of arm condition (Figure 3A, middle panel) with clusters covering right (pre-)motor cortex and bilateral pre-SMA, and left pre-motor cortex. Additionally, a significant interaction was found between body side and arm condition (Figure 3A, right panel) in right (pre-)motor cortex and bilateral SMA; indicating that any cortical differences between right and left leg movement were dependent on whether arms were crossed or swinging, and that any differences in cortical activity between arm conditions were dependent upon the movement of each body side. MNI (Montreal Neurological Institute) coordinates of locations with peak activation and corresponding statistics are listed in Table 2.

**Table 2:** Locations of peak activation for the statistical tests performed for the beamformer contrasts between early DS and SW phase in the beta band

	Region	MNI location of peak activation (mm)	*F*-value	*p*-value
ANOVA main effect body side	anterior cingulate cortex	4, 10, 32	22.37	<0.001
	left (pre-)motor cortex	−44, 6, 34	16.41	<0.001
	right (pre-)motor cortex	34, −6, 48	15.55	<0.001
ANOVA main effect arm condition	right (pre-)motor cortex and bilateral pre-SMA	52, −12, 56	10.42	<0.01
	left pre-motor cortex	−34, 0, 52	10.48	<0.01
ANOVA interaction body side × arm condition	right (pre-)motor cortex	24, 2, 52	23.04	<0.001
	bilateral SMA	−8, 8, 54	10.73	<0.001
Post-hoc left leg *arms crossed* vs. *arms swinging*	right (pre-)motor cortex and bilateral SMA	24, 2, 52	4.91	<0.001
Post-hoc right leg *arms crossed* vs. *arms swinging*	left pre-motor cortex	−40, 2, 56	2.71	<0.01

Further post-hoc comparisons between *arms swinging* and *arms crossed* revealed an increase of beta activity in the *arms crossed* condition for both left and right leg contrasts (i.e., R/L early DS and SW; Figure 3B). For the left leg contrast, this activity was greater in a large cluster covering right (pre-)motor cortex and bilateral SMA, and in the left pre-motor cortex for the right leg contrast. As mentioned for the sensor-level results and as can be seen from the significant source level clusters in Figure 1, these post-hoc comparisons reflect the greater lateralization in beta activity for *arms swinging* compared to *arms crossed*.

#### Gamma frequency band

Differential activations in the gamma band were most prominent when comparing the phases around the HS events (Figure 2, lower row). For the contrasts between early DS and SW phases for each leg, please see Figure S2. For the HS events, we found significant gamma activity in both walking conditions in the medial part of M1 (*p* < 0.001), corresponding with the putative leg representation. This gamma activity was more pronounced at the LHS in the *arms crossed* condition with an extension of activity from M1 toward SMA (Figure 2B).

**Figure 4:**
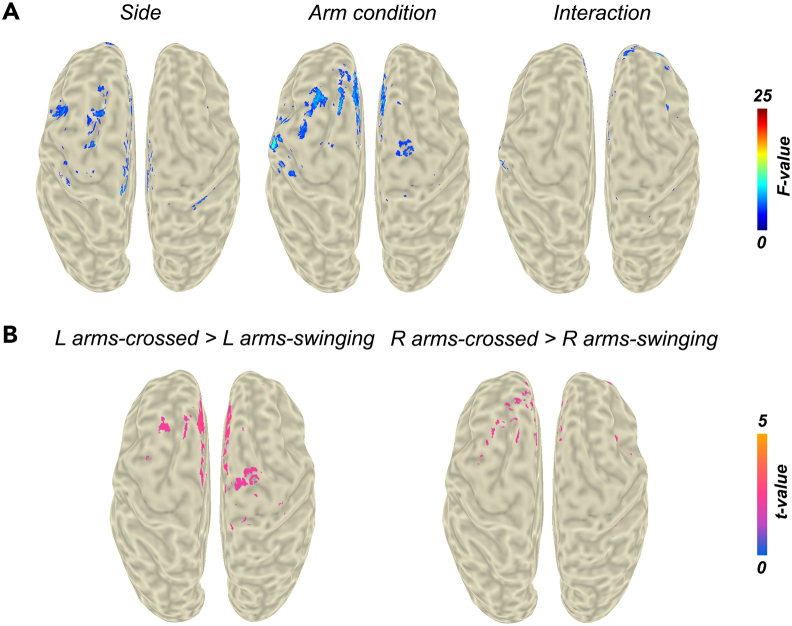
Statistical analysis of the gamma band beamforming contrasts around the HS event Results indicate significant differences in the magnitude of the contrast around the HS event. (A) Two-way ANOVA with side (left vs. right leg) and arm condition (*arms swinging* vs. *arms crossed* condition) as the main factors. The maps were thresholded at *p* < 0.05. (B) Post-hoc *t*-test for the main effect of arm condition. The maps were thresholded at *p* < 0.025, adjusted alpha level for two comparisons. HS, heel-strike; R, right; L, left.

The subsequent ANOVA revealed a significant main effect of body side (Figure 4A, left) in the leg area of M1 and in the left pre-motor cortex, and a significant main effect of arm condition (Figure 4A, middle) in bilateral pre-SMA, left sensorimotor cortex, and left frontal cortex. Additionally, the results showed a small cluster of significant activity in right pre-motor cortex and further scattered activity that reached significance level. However, since we did not correct for multiple comparisons, these results should be interpreted with caution. Likewise, no prominent significant activity was detected for the interaction effect. MNI coordinates of locations with peak activation and corresponding statistics are listed in Table 3.

**Table 3:** Locations of peak activation for the statistical tests performed for the beamformer contrasts around the HS event in the gamma band

	Region	MNI location of peak activation (mm)	*F*-value	*p*-value
ANOVA main effect body side	leg area of M1	−4, −40, 78	8.75	<0.01
	left pre-motor cortex	−28, 18, 34	8.30	<0.01
ANOVA main effect arm condition	bilateral pre-SMA	−4, 28, 46	13.74	<0.01
	left sensorimotor cortex	−50, −16, 30	18.82	<0.001
	left frontal cortex	−22, 20, 64	8.92	<0.01
	right pre-motor cortex	22, −14, 58	6.15	<0.05
Post-hoc left leg *arms crossed* vs. *arms swinging*	bilateral (pre-)SMA	−4, 28, 46	3.32	<0.01
Post-hoc right leg *arms crossed* vs. *arms swinging*	bilateral (pre-)SMA	−4, 30, 46	3.24	<0.01

Further post-hoc analysis comparing *arms swinging* and *arms crossed* (Figure 4B) resulted in an increase of gamma activity in bilateral (pre-)SMA in the *arms crossed* condition for both the left and right leg contrast.

## Discussion

In this study, we demonstrated that cortical activity differs between distinct phases of the gait cycle and when participants walk with or without arm swing. In walking with both arms swinging, we found significant differences in beta power between the DS and SW phases in sensorimotor cortex ipsilateral to the leading leg. This lateralization disappeared when participants walked without arm swing, which was dominated by activity in bilateral SMA and frontal cortex. In addition, we found a significant difference in gamma power before and after the time of HS. The differences were observed in the medial part of M1, the putative leg area, irrespective of the arms swinging or being crossed.

At the sensor level, our results showed clear ERD-ERS modulations during the gait cycle in both conditions. This was especially pronounced in the beta band showing peak power right after each of the two HS events, i.e., in the early DS phases. Notably, in the *arms swinging* condition, the beta ERS at the HS events demonstrated a lateralization effect over the electrodes close to the cortical representation of arm movement in M1 (i.e., C3 and C4). The lateralization disappeared when both arms were crossed, suggesting a role for beta activity in swinging of the arms during gait. The observed ERD-ERS beta modulations resemble the typical desynchronization that occurs during the preparation and execution of a limb or finger movement, followed by a synchronization rebound after movement termination.^26^^,^^27^ Our results suggest that this notion may also be true in gait, as at the HS, both arms have reached their maximum forward and backward position and both legs are in contact with the ground. Therefore, the observed power differences at the HS events in those electrodes may be attributed to the transition between different muscle activations in the gait cycle, in this case the end of the forward movement of the contralateral arm and the end of backward movement of the ipsilateral arm.

Consistent with our findings at the sensor level, the source level contrasts between early DS and SW phases in the beta band revealed a significant lateralization in the arm area of the primary sensorimotor cortex with greater activation ipsilateral to the leading leg in the *arms swinging* condition. Such left-right lateralization of beta modulations during gait has been observed previously by others where ERS typically seems to appear greatest after the SW phase of the ipsilateral leg, while greatest ERD appears either during the SW phase of the contralateral leg,^13^^,^^21^^,^^28^ during the swing phase of the left leg,^11^^,^^29^ or without a clear lateralization pattern.^19^ Our beamforming contrasts were based on the sequential ERD-ERS order typically observed for movement-related activity,^26^ therefore offering an alternative perspective on the lateralization of beta modulations compared to previous literature. It is encouraging that, despite differences in source localization methods and choice of contrast windows, the lateralization of beta modulations during the gait cycle holds as a robust finding. Most importantly, in our study, the lateralization disappeared while an increase of activity emerged in the sensorimotor and premotor cortex contralateral to the leading leg when arms were constrained. The disappearance of the lateralization was hence clearly related to arm movement.

While activity in M1 is typically associated with control of the contralateral limb, we cannot rule out the possibility of ipsilateral contributions as both limbs are moving at the same time. In previous literature, cortical activity ipsilateral to the moving limb has been suggested to be related to planning and execution of (complex) movement.^30^^,^^31^^,^^32^^,^^33^^,^^34^ Notably, it has been shown that lower limb movement has greater M1 activity ipsilateral to a moving limb with less lateralized activity compared to upper limb movement.^35^^,^^36^ Moreover, studies using electromyography have shown ipsilateral coupling between arm and leg muscles,^3^^,^^37^^,^^38^ and a significant positive correlation between activity in premotor cortex and ipsilateral leg muscles during walking.^39^ In light of these findings, the role of the ipsilateral hemisphere in our study may be associated with the neural coupling between the arms and legs during walking.

Arm swing has been shown to influence human gait stability.^40^^,^^41^ An increase in beta power in the left premotor cortex has been found in a more stable walking condition in healthy individuals,^17^ while absence of arm swing, both in healthy participants and individuals with Parkinson’s disease, has been associated with a reduction of beta power over SMA.^12^^,^^25^ Our results therefore suggest that the increased activity in SMA when crossing the arms, may reflect a compensation strategy for a loss of gait stability due to the absence of arm swing. Furthermore, previous studies have shown that upper and lower limbs are coupled through common neural inputs,^37^^,^^42^^,^^43^^,^^44^ and that the upper limb muscles additionally drive lower limb muscles through neural connections in which corticospinal pathways are involved.^3^^,^^4^^,^^45^ Beta activity appears to have a pivotal role as a common driver in the coordination of interlimb movement as synchronization between cortex and muscles has been reported primarily in this frequency band.^46^^,^^47^^,^^48^^,^^49^ In line with this, previous studies using intermuscular coherence showed stronger coupling between shoulder and proximal muscles than the distal muscles in the beta band and found that shoulder muscles drive the leg muscles.^3^^,^^37^ This suggests that beta activity may provide the neural substrate for the facilitatory role of arm swing on lower limb movements during gait, and stabilization of the gait pattern in general.

Our study indicates differential contributions of anterior and posterior cingulate cortex during the gait cycle. In the *arms swinging* condition, when comparing the right early DS and SW phases, we observed beta activity in posterior cingulate cortex, while this activity emerged more anteriorly when comparing the right early DS with late SW phase (see Figure S1 in comparison with Figure 1). In other words, beta activity shifted from posterior to anterior cingulate cortex between early and late SW phases. The anterior cingulate cortex is suggested to play an important role in motor control, including preparation and execution of motor commands, planning, and error detection and correction,^50^^,^^51^^,^^52^ while the posterior cingulate cortex is proposed to be involved in spatial orientation and navigation.^14^^,^^53^^,^^54^ In gait-related studies, an increase in beta activity in anterior cingulate cortex prior to HS has been reported by Gwin and colleagues.^9^ The authors suggested that activation of the anterior cingulate cortex may be related to error detection and correction prior to foot placement. The posterior cingulate cortex on the other hand has been mainly reported in the theta band in the concept of balance and perturbation.^14^ However, a recent study on the comparison between walking with and without ankle exoskeleton, found an increase of alpha and beta power modulation in posterior cingulate cortex during IDS (RHS-LTO), and a decrease of alpha and beta power modulation during RSW phase when walking with activation of ankle exoskeleton.^55^ In our study, the absence of posterior cingulate activation when comparing DS and SW phases in the *arms crossed* condition suggests that this differences between early and late swing phase may be related to the swinging of the arms. Using a recumbent stepper, Kline and colleagues^56^ obtained similar results by showing that activation of posterior cingulate cortex was only observed in the conditions when subjects exercised with only arms, or arms and legs together, but not in the condition when only the legs were active. Taken together, our results are in line with previous studies suggesting that walking with arm swing is under more cortical control, where the activation of posterior cingulate cortex in the early swing phase may be related to the spatial orientation of the arms during walking.

Consistent with previous literature,^12^^,^^57^ we observed gait-cycle dependent modulations in the gamma band in both walking conditions. The observed sensor-level increase in gamma power in the *arms crossed* condition for the electrodes covering central sensorimotor cortex is also in line with previous work.^12^ Our source level analysis subsequently revealed a significant gamma power increase around the time of HS in the medial part of M1, the presumed leg area, irrespective of the arms swinging or being crossed. Instead, gamma power in bilateral (pre-)SMA increased at the HS in the absence of arm swing. The contribution of motor cortex to ankle muscles prior to HS and during the stance phase in the gamma band has been reported in previous studies.^58^^,^^59^ The increase in bilateral (pre-)SMA in the absence of arms swinging might be a compensation strategy to maintain the coordinated cyclic movement pattern of the legs; however, we are not able to rule out an increased contribution of the trunk either. Our results at least further corroborate involvement of the gamma band in the coordination of leg movement during gait.^13^^,^^57^

### Limitations of the study

An inherent limitation of studying the contribution of arm swing to the cortical control of gait is the impossibility to completely eliminate the influence of the arms on the gait pattern in a control condition. While asking participants to walk with arms crossed removes large-amplitude motion of the arms, it might simultaneously alter the way in which the resulting gait pattern is controlled. Similarly, it remains difficult to attribute contralateral and ipsilateral beta activity to individual limbs in the current experimental design. Future studies could benefit from including an arm swing only condition (without leg movement) or by constraining only one arm at a time during walking to further disentangle limb-specific activity patterns. Furthermore, although the sample size in our study was comparable to that of most previous gait-related EEG studies,^9^^,^^10^^,^^16^^,^^17^^,^^19^^,^^20^^,^^21^^,^^23^^,^^28^^,^^29^^,^^60^ it was lower compared to others.^11^^,^^12^^,^^13^^,^^14^^,^^15^^,^^18^ Together with the relatively low number of gait cycles that were available for each participant, this might have lowered the statistical power and may explain why not all ANOVA effects survived a voxel-level multiple comparisons correction.

## STAR★Methods

### Key resources table

**Table:** 

REAGENT or RESOURCE	SOURCE	IDENTIFIER
**Software and algorithms**		

MATLAB 2018b	MathWorks	www.mathworks.com
FieldTrip	FieldTrip	www.fieldtriptoolbox.org
SPM12	SPM12	www.fil.ion.ucl.ac.uk/spm/software/spm12

### Resource availability

#### Lead contact

Further information and requests for resources should be directed to and will be fulfilled by the lead contact, dr. Bernadette C. M. van Wijk (b.c.m.van.wijk@vu.nl).

#### Materials availability

This study did not generate new unique reagents.

#### Data and code availability

• Data reported in this paper will be shared by the lead contact upon reasonable request.
• Code will be shared by the lead contact upon reasonable request.
• Any additional information required to reanalyze the data reported in this paper is available from the lead contact upon reasonable request.

### Experimental model and study participant details

#### Participants

Twelve healthy young adults (24.5 ± 2.3 years [mean ± SD], two male, four left-handed, all Western European) participated in the experiment. The study was conducted in accordance with the guidelines of the Declaration of Helsinki. Prior to the experiment, all procedures were approved by the Scientific and Ethical Review Board of the Faculty of Human Movement Sciences, Vrije Universiteit Amsterdam (VCWE-2017-132). All participants read and signed a written informed consent form.

### Method details

#### Protocol and data acquisition

Participants were invited to walk on an instrumented dual-belt treadmill (Motek Medical BV, Amsterdam, the Netherlands) at three different speeds (3, 3.5, 4 km/h), each around 35 s in two conditions: (1) with arms swinging normally and (2) with arms crossed across the chest. These conditions will be referred to as *arms swinging* and *arms crossed*. The order of speeds and types of walking was randomized across participants. In total, six trials (3 speeds × 2 conditions) were used for each participant for further processing. Ground reaction forces during walking were recorded with the force plate embedded in the treadmill. In addition, full-body kinematics were recorded (Optotrak, Northern Digital, Waterloo ON, Canada) with five cluster markers attached on the upper and lower arms, upper and lower legs, and on the heel. Kinematic and force plate data were sampled at 70 Hz and served to detect the moments of HS TO. As part of a larger study, electromyography was also collected and has already been reported together with the kinematics.^37^ Here, we focus on the EEG measurements that were collected with a TMSi Refa amplifier (Twente Medical Systems International, the Netherlands) with 64 sintered Ag/AgCl electrodes with common average reference. Electrode impedances were kept below 20 kΩ. EEG data were sampled at a rate of 2048 Hz. Kinematics, ground reaction force and EEG data were synchronized online.

### Quantification and statistical analysis

#### Electroencephalography preprocessing

EEG signals were preprocessed offline using MATLAB (Mathworks Inc., Natick, USA) through a pipeline utilizing the FieldTrip toolbox, version 20210325.^61^ For each trial, first, individual channels were identified as ‘bad’ in case the channel contained a flat line or an excessive mean or standard deviation in terms of amplitude (>1e6). Whenever appropriate, these EEG channels were interpolated by their neighboring channels using spherical splines. Signals were band-pass filtered between 5 and 200 Hz using a bidirectional second-order Butterworth filter. To remove the line noise and its harmonics, we notch-filtered at 50, 100, 150, and 200 Hz using a windowed-sinc FIR filter. Muscle and eye movement artifacts were reduced via independent component analysis (fastICA^62^) by following our previous approach.^17^^,^^63^ Mode removal was based on (1) the corresponding spectral distribution that served to identify electromyographic activity and movement artifacts (modes with median frequency >100 Hz or <2 Hz), and (2) the modes’ topography to identify exaggerated eye movements and muscle activity (channels Fp1, Fpz, Fp2, F7, F8, FT7, FT8 and O1, Oz, O2, T7, T8, M1, M2, respectively). On average 29 ± 4 (mean ± SD) modes were removed per subject (range: 13–41). The remaining independent components were projected back to the original electrode space and re-referenced using an average reference across all channels.

#### Gait event detection

Right/left heel-strike and toe-off events (RHS/LHS and RTO/LTO, respectively) were detected offline from the force plate data. HS and TO-events were defined as the first sample crossing the threshold of 8% of the average vertical ground reaction force during the trial. These event moments were verified by comparing with HS and TO as identified in the kinematic data; see Kerkman et al.^37^ for details.

#### Electroencephalography analysis

##### Time-frequency analysis at sensor level

To estimate event-related spectral power, trials were segmented to full strides starting from RHS plus a time buffer of around 1 s for spectral calculations. On average we encountered 61 ± 5 (mean ± SD) gait cycles per participant and condition. Morlet wavelets with 4.5 cycles served as time-frequency estimators for frequencies between 3 and 90 Hz in steps of 0.25 Hz. To account for variability in walking speeds and stride durations, the gait cycles were time normalized using linear interpolation, such that the intervals between the individual gait events obtained an equal time point in the resulting time-frequency spectra. These time points were set by the averages of gait-event moments across strides and participants and resulted in: (RHS-LTO-LHS-RTO-RHS) = (0-15-50-65-100% of the gait cycle). To highlight the gait-related modulation in spectral power, we log transformed the wavelet power and subtracted the mean over the stride time at every frequency (bin). This served as a proxy for the relative power changes as a function of time and frequency. Next, we computed the mean relative log wavelet power within the beta (15–30 Hz) and gamma (30–50 Hz) frequency bands for channels Cz, C3, C4, and Fz for putative leg, right and left arm representations in M1, and for the SMA, respectively. Henceforth, we refer to these as average beta/gamma wavelet power. This analysis at the sensor level for the specified electrodes served as an initial analysis to visualize the beta changes related to arm swing movement that were reported in previous studies.^12^^,^^25^

##### Source localization

To reconstruct the cortical sources of beta and gamma band activity, we applied DICS beamforming^64^ based on the full set of 64 EEG electrodes. Unlike the combination of ICA with dipole fitting approaches that have been frequently applied in gait-related EEG studies,^9^^,^^11^^,^^14^^,^^16^^,^^19^^,^^21^^,^^29^^,^^60^ beamforming does not rely on a predetermined selection of the number of sources that is concurrently active.^65^ Instead, for each voxel in source space, a spatial filter is computed that preserves activity estimated by the lead-field while minimizing signal leakage from other sources. DICS beamforming has been previously used by our research group to localize gait-related beta band EEG activity.^17^^,^^63^ The forward solution was constructed using a five-compartment (scalp, skull, scalp, gray matter, white matter, cerebrospinal fluid) finite element model using the integrated FieldTrip-SimBio toolbox^66^ based on FieldTrip’s 2020 template MRI in MNI coordinates with conductivity values as defined by Birot et al.^67^ EEG electrodes were aligned to this head model and the corresponding lead-field matrix was computed at a 2 mm resolution. Spectral power and cross-spectral densities were estimated using the same (time-normalized) wavelet-based approach as outlined above without log transformation. For both conditions (*arms swinging* and *arms crossed*) together, a common spatial filter was estimated for beta and gamma frequency bands separately based on the full stride with the regularization parameter set to 5%. Next, for each condition separately, we extracted the four phases of the gait cycle: right and left swing (SW) and the following DS phases. SW refers to the period between TO and HS, while the double DS refers to the period between HS and TO. To differentiate the right and left DS phases following the right and left HS, hereafter, we use the terms initial DS (IDS), and final DS (FDS) respectively. We divided the four phases in half to obtain the early (first half), and late (second half) SW and DS phases (see Figure 1, top panel). The common filter was used for projecting the average power in the individual phases to source level. Hence, a total of 16 source images per participant (right/left, early/late, DS/SW, arms swinging/arms crossed) entered subsequent statistical assessments.

#### Statistics

##### Gait characteristics

We determined the mean, standard deviation, and coefficient of variation of the stride duration and the duration of the individual phases of the gait cycles and compared them between walking conditions using paired *t*-tests.

##### Source level statistics

Prior to computing the dependent samples *t*-statistics, power values were log-transformed to stabilize variance. Differences in beamformer power between different phases in the gait cycle were analyzed via cluster-based permutation testing.^68^^,^^69^ To address the multiple comparisons problem at the voxel level, cluster-level statistics were obtained by summing the *t*-values within each cluster. The cluster-level statistic with the highest value then served as test statistic, and the probabilities were estimated using FieldTrip’s Monte Carlo method. The cluster distribution was estimated via 2^12^ permutations and clusters were thresholded at α_cluster_ = 0.01. Beamformer contrasts (*t*-values) were computed in two stages. First, ratios between early DS and early SW phases were calculated for each leg and each condition separately (Figure 1). This analysis aimed to capture cortical differences between early DS relative to early SW phases of the same leg (end of the step versus SW phase), where we observed the greatest beta ERS and ERD, respectively. Second, additional contrasts were computed between early DS and late SW phases, focusing on changes around HS events (Figure 2) where adjustments in limb deceleration and preparation for stance are crucial. All contrasts were computed for beta and gamma frequencies separately. Correction for multiple comparisons for the number of contrasts (right/left, arms swinging/arms crossed) was performed with the Bonferroni method (*p* = 0.05/4 = 0.0125).

Next to these separate *t*-tests, to see if the observed difference was related to arm movement, we also conducted a two-way ANOVA (left/right phase differences × arm condition) with repeated measures using SPM (version 12; http://www.fil.ion.ucl.ac.uk/spm/software/spm12/). In view of the *t*-test results above, here we used the differences between log-transformed power values of DS and SW and normalized them by their sum. These ‘pseudo-*t*-values’ were subsequently Fisher *z*-transformed to stabilize variance (cf. above). The statistical threshold was set at *p* < 0.05, with no correction for multiple comparisons at the voxel level. Further post-hoc analysis was also performed with correction for multiple comparisons for the number of contrasts using the Bonferroni method (*p* = 0.05/2 = 0.025). In the final step, we used the SPM Anatomy Toolbox (version 3.0, Forschungszentrum Jülich GmbH; RRID:SCR_013273) to match significant peak activations with the anatomical probability map.^70^^,^^71^^,^^72^

A Region of Interest mask was applied on the statistical outputs to specifically investigate the effects in regions that are commonly activated during human locomotion and included the frontal, cingulate, and sensorimotor cortex. The temporal, parietal and occipital cortices were masked out due to the possible interference from scalp and neck muscle activations during gait.
