# Supplementary material for: Lateralized modulation of cortical beta power during human gait is related to arm swing
Source: iScience. 2024 Jun 17;27(7):110301. doi: 10.1016/j.isci.2024.110301 (PMC11269954; doi:10.1016/j.isci.2024.110301)
Supplement: Document S1. Figures S1 and S2 [file mmc1.pdf]

iScience, Volume *27*

Supplemental information

Lateralized modulation of cortical beta power during human gait is related to arm swing

Marzieh Borhanazad, Bernadette C.M. van Wijk, Annemieke I. Buizer, Jennifer N. Kerkman, Annike Bekius, Nadia Dominici, and Andreas Daffertshofer

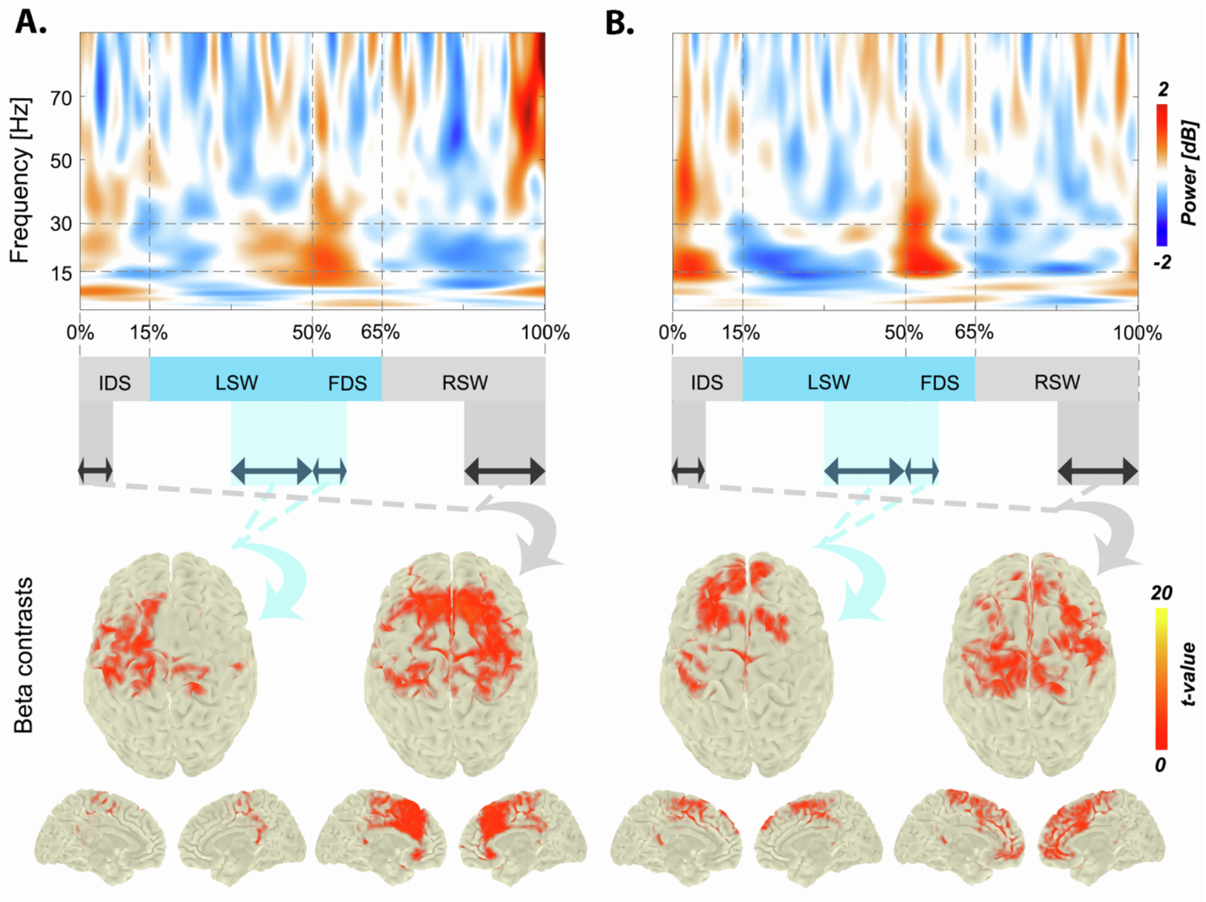

Figure S1. Time-frequency analysis and beamforming contrasts around the HS event for the beta band, related to Figure 1. A) *Arms swinging* condition. B) *Arms crossed* condition. The time-frequency representation for electrode C3 is illustrated. The statistical contrasts between beta beamformers (based on all 64 electrodes) in early DS and late SW phase (around HS event) for each leg are shown at the bottom of the figure. The horizontal arrows indicate early (first half) DS and late (second half) SW phase. *T*-values for voxels belonging to significant clusters ($p < 0.0125$; adjusted alpha level for four comparisons) are displayed after anatomical masking. By comparing early DS and late SW phases in the *arms swinging* condition, we found significant activity in M1 and in premotor cortex in the hemisphere ipsilateral to the moving leg ($p < 0.001$). Only for the contrast on the right leg, significant activity ($p < 0.001$) was observed in bilateral SMA, and anterior cingulate cortex. In the *arms crossed* condition, however, the lateralized pattern was replaced by symmetric activity in M1 and frontal cortex ($p < 0.001$). Moreover, only for the right leg contrast, activity was also observed in the cingulate cortex but towards the anterior part. HS, heel-strike; DS, double support; SW, swing; IDS, initial double support; LSW, left swing; FDS, final double support; RSW, right swing.

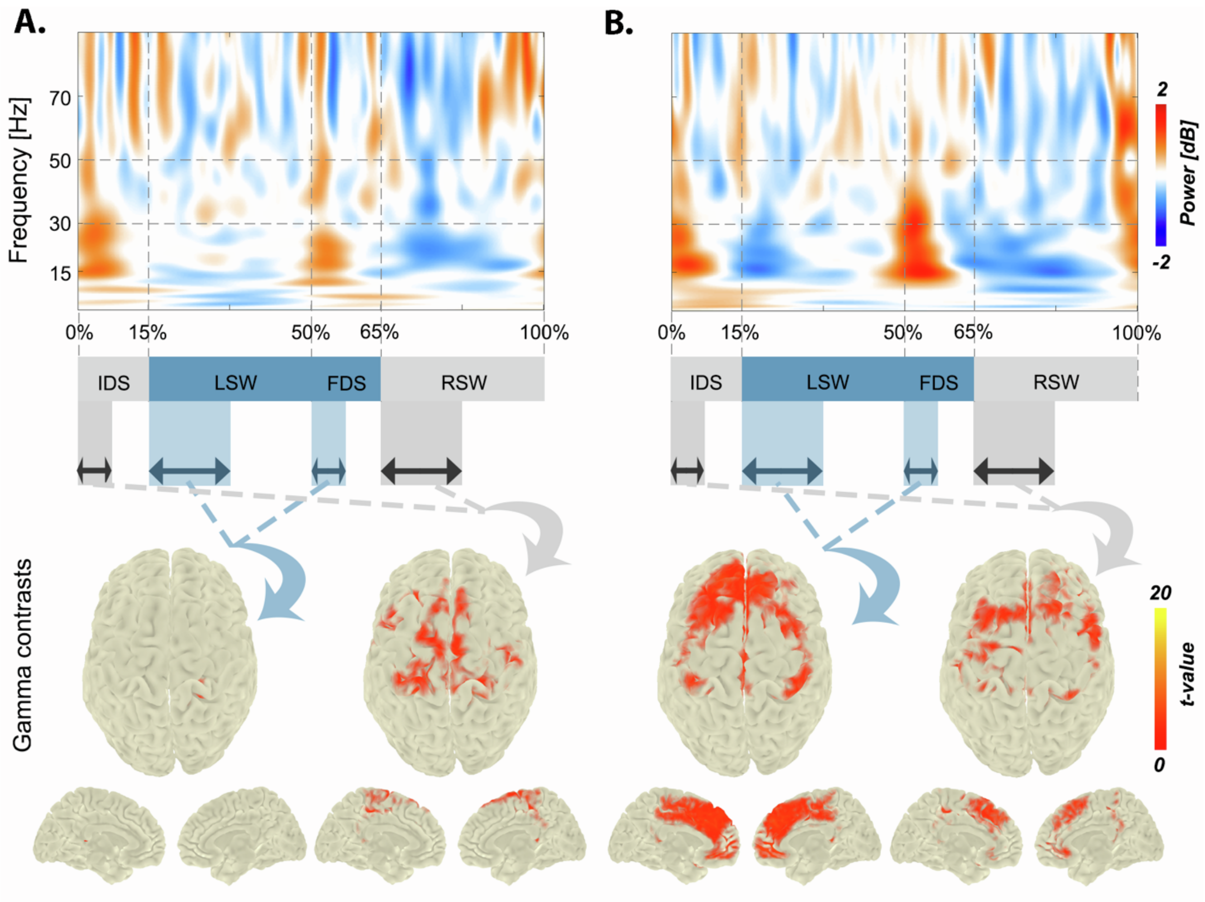

Figure S2. Time-frequency analysis and beamforming contrasts between early DS and SW phases in the gamma band, related to Figure 2. A) *Arms swinging* condition. B) *Arms crossed* condition. The time-frequency representation for electrode C4 is illustrated. The statistical contrasts between gamma beamformers (based on all 64 electrodes) in early DS and SW phases for each leg are shown at the bottom of the figures. The horizontal arrows indicate early (first half) DS and late (second half) SW phase. *T*-values for voxels belonging to significant clusters ($p < 0.0125$; adjusted alpha level for four comparisons) are displayed after anatomical masking. The resulting contrast between early DS and SW phases, showed an increase of gamma activity in bilateral SMA in the *arms crossed* condition ($p < 0.001$). This gamma activity was more pronounced in the left leg contrast in the *arms crossed* condition with an extension of activity towards the pre-frontal and anterior cingulate cortex. DS, double support; SW, swing; IDS, initial double support; LSW, left swing; FDS, final double support; RSW, right swing.
